# Uses of quick response codes in healthcare education: a scoping review

**DOI:** 10.1186/s12909-019-1876-4

**Published:** 2019-12-06

**Authors:** Chiraag Thakrar Karia, Andrew Hughes, Sue Carr

**Affiliations:** Department of Clinical Education, University Hospitals of Leicester, Leicester, LE1 5WW UK

**Keywords:** Quick response codes, Technology enhanced learning, MedTech

## Abstract

**Background:**

To review published literature on the use of quick response (QR) codes within healthcare education. In addition, the authors aimed to gain information on user perceptions and the challenges faced when implementing QR codes in an educational context.

**Methods:**

Medline, EMBASE, CINAHL (Cumulative Index to Nursing and Allied Health Literature), HMIC (Healthcare Management Information Consortium) and HBE (Health Business Elite) were searched using specified search terms that included ‘quick response code’ and ‘education’. Title and abstract review of 217 publications was performed. Papers which discussed the application of QR codes relevant to healthcare education were included. A total of 24 articles were reviewed and thematic analysis conducted to generate themes.

**Results:**

Use of QR codes in healthcare education were broadly aligned to four common themes. These included: to increase participant engagement, for simulation training, for just-in-time (JIT) learning and to facilitate with administrative tasks in training. Perceptions towards the use of QR codes was generally positive. Challenges identified, included: problems with technical infrastructure, unavailability of smartphones and resistance to use in certain environments.

**Conclusions:**

The use of QR codes for healthcare education is increasing, and whilst they offer some advantages there are also some important considerations including: provision of the necessary technological infrastructure, patient and staff safety and governance and adherence to guidelines on safe and appropriate use of this technology in sensitive settings.

## Background

In 1994, quick response (QR) codes were developed by Denso Wave, a previous subsidiary company of Toyota, to track car components during manufacturing and distribution [1]. Denso Wave holds the patent for QR codes but has made the technology widely available, free of cost. Since then, the use of these black-and-white pixelated squares has rapidly increased, due to their ability to contain more information than a standard bar code in a 10th of the space and their high-speed, omnidirectional scanning capabilities.

QR codes can be easily created online from a range of websites. They can link to simple text, a website, a template email or text message, make a phone call, show a geographical location and access a PDF or specific application on a mobile device [2]. The increased availability of smartphones with cameras has led to QR codes being applied to a wide range of commercial applications including: marketing [3], ticket management in transportation [4] and more recently, social media applications such as Snapchat [5].

The versatility of QR codes has also generated interest for use in a number of healthcare settings. Applications have been explored for storage of case histories in maxillofacial radiology [6], safer use of medications by elderly patients [7] and patient instructions following orthopaedic cast application [8].

In addition, QR codes have been shown to have value in education. Technology now facilitates a greater variety of visual learning materials. Integration of verbal and visual learning materials in spatial contiguity reduces the split-attention effect [9]. In addition, QR codes could offer a method to temporally link a variety of materials reducing learners’ extraneous cognitive load. QR codes can be thought of as advocating a student-centred learning approach by giving students’ autonomy over what and how they learn [10]. QR codes can help to enhance students’ intrinsic motivation and allow accommodation of different learning preferences by providing enrichment of current learning materials, allowing flexibility for accessing materials and offering self-assessment opportunities [11]. Through the paradigm of social constructivism, QR codes are enablers of learning through interaction as demonstrated in studies by Al-Khalifa [12] and Chaisatien and Akahori [13]. Specific examples are detailed below.

In the classroom, Rikala & Kankaanranta demonstrated that QR codes support both independent and collaborative learning approaches and can engage learners [14]. Lai et al. demonstrated how QR code incorporation onto green maps [15] could help to achieve learning outcomes, provide opportunities for further interaction and allow for teaching in a diverse range of locations. Lee et al. used QR codes during a biology field trip [16] and postulated that it could more effectively motivate learners than non-digital means in today’s “information” generation. The potential for healthcare professional education holds equally promising potential, but literature on this topic has not yet been reviewed or mapped.

In this review, we aimed to explore the use of QR codes specifically for healthcare education, identify perceptions towards their use and any challenges that have been encountered during implementation.

## Methods

The review followed the PRISMA (preferred reporting items for systematic reviews and meta-analyses) extension for scoping reviews checklist and explanation [17]. Arksey and O’Malley’s six steps were used as the methodological framework [18].

### Research questions

The research questions that guided this review were:

1. How are QR codes currently being used in healthcare education?
2. What are the perceptions of individuals that currently use QR codes for healthcare education?
3. Have challenges been identified when implementing QR codes for healthcare education?

### Identification of relevant studies

Databases used for the search were Medline, EMBASE, CINAHL (Cumulative Index to Nursing and Allied Health Literature), HMIC (Healthcare Management Information Consortium) and HBE (Health Business Elite). These were selected following discussion with a clinical librarian to identify those most likely to produce the greatest number of relevant articles.

Database searches were undertaken on 8 February 2019. The following keywords were used: qr. ADJ3 cod*, (quick response) AND ((barcod*) OR (cod*)), matrix ADJ3 barcod*, ((two-dimensional) OR (2d ADJ3 barcod*) OR (cod*)), ((Educat*) OR (train*) or (instruct*). MeSH (Medical Subject Headings) terms included: medical education, professional education, clinical competence and inservice training. These keywords and MeSH terms were combined with Boolean operators to yield relevant results.

The search was conducted without time or language restrictions. A total of 185 records were obtained. Additional records were identified through other sources, such as Google Scholar and reference mining, bringing the total number of records to 217.

### Study selection

All articles were initially screened for suitability from the title by CTK and AH. Articles were included if they discussed an application of QR codes (*n* = 110). Abstracts were subsequently and independently reviewed for eligibility by CTK and AH, before a further review of the full article. Records were excluded if the main application of QR codes was not for healthcare education (*n* = 57) or if it did not discuss an original application for QR code use (*n* = 29). Any disagreement over article eligibility was resolved through further discussion between CTK and AH.

**Fig. 1:**
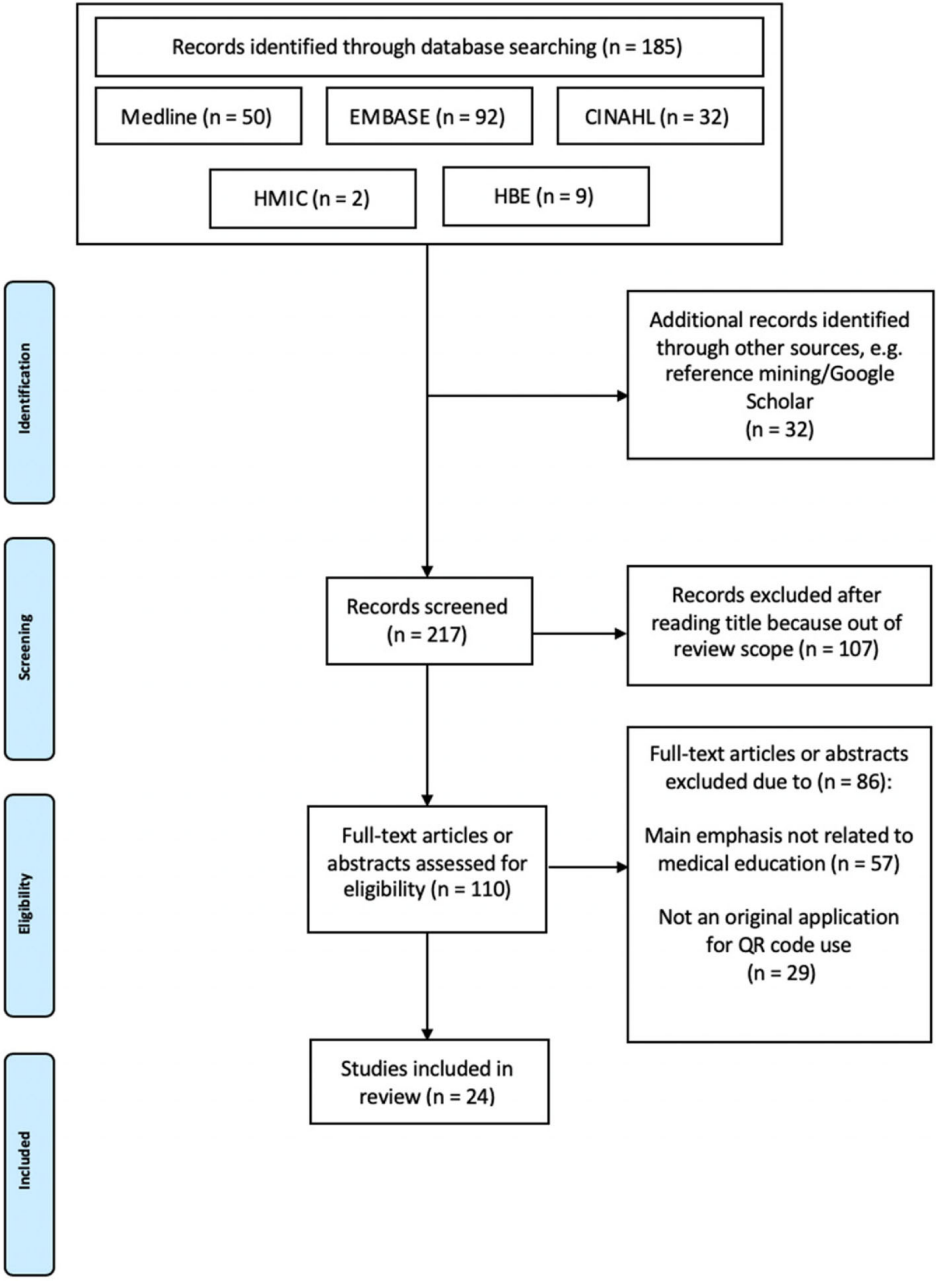
PRISMA Flowchart

The PRISMA flowchart was used to show the search strategy and subsequent study selection process (Fig. 1). Citations and bibliographies were managed using the software package Mendeley Desktop for macOS (Mendeley Ltd., London, UK).

### Charting, collating and reporting of the studies

A data-charting form was created by CTK and AH on an Excel spreadsheet using Microsoft Excel for Mac Version 16.25 (Microsoft Corp., Redmond, WA, USA). Data concerning study design, methods, participants, the intervention and outcomes were extracted independently from the articles by CTK and AH. This was followed by narrative information on problems associated with the application of QR codes. A further review of the form was performed thereafter to ensure consistency and agreement on extraction of relevant information.

Narrative data synthesis was conducted using thematic analysis. This was performed by CTK, AH and SC using content analysis to categorise, code and sort articles on QR codes in healthcare education into distinct uses. Any conflicts over coding of articles was resolved through further discussion between CTK and SC.

## Results

### Article characteristics

A total of 24 articles published between 2011 and 2019 were included in this review. Most studies took place in Western countries (United States being the major contributor (*n* = 11)). The majority of articles reported the use of QR codes for nursing students (*n* = 6). Article types included research articles, editorials, communications and commentaries. A summary of article characteristics is show in in Table 1.

**Table 1:** Summary of article characteristics

	n, (total = 24)	%
Publication Year		
2011–2013	3	13
2014–2015	4	17
2016–2017	12	50
2018–2019	5	21
Publication Type		
Commentary	2	8
Communication	1	4
Editorial	1	4
Research	20	83
Study Location		
Australia	1	4
Canada	1	4
Germany	3	13
Israel	1	4
Philippines	1	4
Singapore	1	4
Taiwan	2	8
United Kingdom	3	13
United States	11	46

The main uses of QR codes in healthcare education emerging from thematic analysis were as follows:

1. Increasing participant engagement
2. Just-in-time (JIT) learning
3. Simulation

4) Training support

Table 2 summarises all articles included in this review.

**Table 2:** Summary of included articles

Author	Users	Aim	Application	Perceptions	Challenges
Avidan et al., 2015 [19]	Anaesthetic trainees	To evaluate the use of QR codes for resident case log management	QR codes containing anaesthesia syllabus data were introduced into an anaesthesia information management system. Codes were automatically generated at the conclusion of each case for resident case logging using a smart device	QR codes were used for case logging after 3 months by 17/26 trainees (65.4%) after 3 months and by 15/25 residents (60%) after 6 months. Usability was rated as good by 9/17 trainees (52.9%) or very good by 7/17 trainees (41.2%) after 3 months. Usability was rated as good by 8/15 trainees (53.3%) or very good by 7/15 trainees (46.7%) after 6 months. Most residents would recommend the use of QR codes for case logging to colleagues who were not using them (At 3 months: 15/17 (88.2%) and at 6 months: 14/15 93.3%). The overall rate of case logging increased from 46.2 to 92.3% after the introduction of QR codes	None identified
Bellot et al., 2015 [20]	Doctor of nursing practice students	To evaluate students’ experiences of using QR codes during a course practicum	Creation of a doctoral-level practicum experience using QR coding to create interactive, individualised patient or provider resource guides	All students reported that creating and scanning QR codes was easy. 83% agreed that QR codes would be of benefit to health care providers and patients in the clinical and community settings. All students stated they were likely to use QR technology in the future	QR codes need to be linked to mobile websites rather than HTML sites to ensure compatibility with mobile devices. Access to the technology necessary to enable full use of a QR-enhanced resource guide
Bukowski et al., 2016 [21]	Nurses	To discuss the structure, current results and future work of an interactive serious game	Gamification of a medication sorting task, in which QR codes were used to identify the simulated patient and their medication requirements	None identified	Difficulty in identification of the QR code in three gaming rounds
Carlson & Gagnon, 2016 [22]	Healthcare students	To test four prototypes of an augmented reality integrated simulation	An augmented reality and interactive storytelling application was used for simulation. This used QR codes placed around the room to trigger prompts, such as videos, locations, diagrams or scripts	Statements regarding the prototypes were rated positively.	Concerns regarding unclear navigation and lack of prompts in some scenarios. Respondents felt the project was not a replacement of simulation
Chang et al., 2018 [23]	Healthcare staff	To describe the development, implementation and initial evaluation of a QR code system integrated within an online leaderboard for cardiopulmonary resuscitation performance	QR codes were used to facilitate a simulation-based cardiopulmonary resuscitation performance leaderboard. After a simulated CPR practice, the participant scanned his or her QR code in order to upload their performance score	The competitive nature of the leaderboard was viewed as divisive	Technical difficulties with the QR code system acted as a barrier to the leaderboard’s longevity. Self-identified “tech averse” were intimidated by the system due to its dependence on coordinating a reliable Wi-Fi and QR code reader application software. Lack of automatic data upload with the QR code system being used as a workaround
Damjanovic et al., 2017 [24]	Medical students	Adaptation of ultrasound simulation software for use in point-of-care ultrasound education to make it affordable and applicable in any simulation setting	QR codes were integrated into training phantoms as part of the simulation training scenarios in order to start displaying video clips	When using QR codes, no additional hardware is required for this simulation in comparison to the use of RFID or NFC tags. Therefore, it can remotely be made available for download	This simulation does not encourage probe manipulation and image optimisation skills in comparison to commercially available systems. Review and quality control of linked teaching materials has to be undertaken along with correct coding for corresponding video clips, which can be time consuming
Downer et al., 2016 [25]	Nursing and midwifery students	To report on a pilot study in which QR codes were used in the clinical laboratory to enhance learning	QR codes were placed on equipment that students were learning how to use and linked to a best-practice videos to remind them how to use the equipment	Students have an “expert” to guide them even when faculty are not available. Students are able to practice skills at a time and place that is convenient for them. When asked their preferred method of access to videos, students commented that QR codes “would probably provide best … and quickest access” as they were “faster and less confusing” and they were the “easiest to access”	None identified
Gardiner et al., 2017 [26]	Plastic surgery trainees	Not applicable	QR codes were used by trainee plastic surgeons to check the latest departmental guidelines. Also, trialing the use of QR codes to deliver patient information leaflets	QR codes have a significant role to play in disseminating information to both patients and colleagues. They are free to create, allow frequent updates, never ‘run out’ and save on the cost and environmental consequences of printing updated leaflets.”	None identified
Jamu et al., 2016 [27]	Nursing staff	To evaluate the effectiveness, acceptability and feasibility of QR codes for multi-professional Just in Time learning	QR Codes were generated for clinical guidelines and policies and positioned at relevant locations on a medical ward for access by nurses as part of the Just in Time learning paradigm	Participants could access pertinent information anytime, anywhere and at the point of need.	Several practitioners in the project had little or no experience with educational technology and did not know their phones were smartphones. There were barriers with accessibility of smartphones and placement of QR codes. Reluctance to use devices in clinical areas or in front of patients.
Lin & Teng, 2018 [28]	Nursing students	To use QR codes to increase engagement in a case-based learning course	QR codes were incorporated into a case-based course to increase student participation and facilitate group discussions during the classes	77% of students gave positive ratings for using QR codes in the case-based learning course. On average, participants agreed most with thestatements “I feel it is easy to use QR codes” and “It’s easier for me to ask questions via QR codes”. Participants at different achievement levels showed different attitudes toward the use of QR Codes.	Difficulty with scanning the QR codes or with slow download speeds
Lin et al., 2017 [29]	Nursing students	To explore student’s viewpoints towards the use of quick response codes in a pharmacology course in Taiwan	QR codes were integrated into a pharmacology literacy course for students to scan during the 7-week course. This was incorporated through problem-based learning activities and in their textbooks	Data were collected using the Nursing Students’ Perceptions of Using QR Codes Scale (NSPQS). Most students agreed that QR codes helped them to learn, considered them motivating and increased their interest in learning. Most of the students considered the use of QR codes to be easy and would not increase their burden of learning. QR codes were stated to be convenient and eco-friendly. Participants liked the idea of using QR codes in the class and would like to use QR codes again.	Challenges included the requirement to internet access or the use of a mobile carrier. Opposition was also encountered due to students stating that looking at a small smart phone screen for long periods would cause eyestrain. Some students did not have a smart phone and were therefore paired with students that had a smart phone. Some students were more accustomed to conventional ways of learning and believed that surfing the internet distracted them from their work.
MacRae, 2011 [30]	Neurosurgery journal readers	To link print content and digital content through the use of smartphones	QR codes are placed on articles in order to connect the reader via smartphone to related videos, figures or tables	None identified	None identified
Mathis et al., 2016 [31]	Medical students	To increase medical student interest in anaesthesiology through creation of a short guide for their clinical rotation	The short guide for a clinical rotation in anaesthesiology included guidance on skills accompanied by QR-code based video illustrations that could be accessed on a smartphone or personal computer.	None identified	None identified
Mogali et al., 2019 [32]	Medical students	To obtain student opinions on the use of QR codes as a learning tool in a medical museum for self- and mobile learning of anatomy and pathology	Used QR codes to enhance medical students experience when visiting an anatomy specimen museum. This was well received by the students and enhanced their exploration experience. Allowed instantaneous access to further information plus ability to save the document to review later.	The majority of students either agreed or strongly agreed that it was easy to access the information about the specimen with QR codes (4.47 ± 0.84), while 96% of students agreed that they were able to correlate the specimen with the annotated images (4.56 ± 0.56). The majority of students (78%) agreed that QR codes were useful for their learning (4.22 ± 0.87), while 75% of students felt QR codes motivated them to visit the Anatomy Resource Centre. Most of the students agreed that QR codes are useful for revision of materials (4.13 ± 1.07) and independent learning (4.38 ± 0.87). These findings suggest that QR codes are not only effective for students learning but also enhance their exploration experience with the museum specimens	QR code-tagged specimens contained sensitive images of human organs and tissues. Therefore, students were reminded that the material was meant for personal use only and not to disseminate the content in any form. The PDF documents linked to QR codes were password protected
Reynolds et al., 2014 [33]	Obstetric-gynaecology trainees / Faculty	To judge the feasibility and acceptability of a novel electronic system for the evaluation of surgical skills	Unique QR codes were added to residents’ ID badges for scanning by faculty in order to provide feedback after surgical procedures.	The evaluation system was quickly accepted by residents and faculty. Thirty (79%) of the 38 indicated it was superior to the previously used handwritten format. 83% stated it provided improved educational benefit and 86^ saw value in continuing this form of resident evaluation. 86% of respondents were satisfied or very satisfied with this format. The electronic system demonstrated improved utilization compared with paper evaluations, with a mean of 23 electronic evaluations submitted per resident during a 6-month period versus 14 paper assessments per resident during an earlier period of 6 months.	Some faculty expressed discomfort with using the electronic format because it was unfamiliar technology
Rosario-Raymundo, 2017 [34]	Nursing staff	To assess the usability, accessibility and feasibility of using QR codes for mobile learning and the factors that impact this	QR codes were generated that linked to a mobile website, connected to a doctor’s telephone number or revealed alphanumeric information to allow for immediate access at the point of care	QR codes demonstrated a high level of functionality, usability and usefulness. The majority of the participants liked the experience of using the QR codes, citing ease of use of the QR codes; a high level of satisfaction in the kind and amount of supplementary medical information accessed; and the favorable effect the QR codes had on their personal learning.	Difficulty in scanning codes from a participant which was attributed to a smart phone with an older operating system and limited data storage. Internet connection speed and good lighting were identified as factors that determined ease of use. One participant disliked the need to download a QR code reader
Shustack, 2018 [35]	Nursing students	Discussed the different uses of QR codes for engaging millennial nursing students	Applications mentioned include their use in the classroom, simulation laboratory and for creating interactive poster displays	None identified	None identified
Siderits et al., 2011 [36]	Tumour board presentation attendees	Describes the use of QR codes in tumour board presentations to distribute educational content	QR codes were included into every presentation to link to the Tumour Board Toolbox and also provided content for histology images or cases	Stated to be an easy and effective way to incorporate more educational content from various sources while allowing audience members to conveniently access, acquire, manipulate and share the information. Provides convenience and thoroughness for the educator	Adding a logo into the QR code can cause “disruption” when attempting to scan
Snyder et al., 2018 [37]	Medical students	To determine whether QR code-linked online feedback forms improve the frequency and efficiency of rater feedback	Clerkship students were provided with laminated cards with a QR code on that was scanned by their preceptor after each learning session to provide feedback. This was a multisite evaluation of the innovation.	The QR feedback method had the highest usability rating when compared to online or paper methods. Accessing feedback via QR code was associated with the shortest time to prepare feedback. QR feedback forms were found to be portable and easily accessible.	Adoption was challenged by limitations in the wireless network at some clerkship sites
Sobhani et al., 2017 [38]	Medical students	To determine if a QR evaluation tool would improve timeliness, usability and the efficacy of giving and receiving of feedback compared to paper evaluations	Smartphone-based evaluations were created that were accessed from a personal QR code given to each student in the intervention group. These were scanned by instructors to provide feedback	Compared to those using paper evaluations, instructors using QR evaluations were significantly more likely to agree that the evaluation tool was easy to understand (100% vs 43%) and easy to navigate (82% vs 57%), and that the evaluation tool was effective in providing feedback (75% versus 29%). Evaluators using QR codes also felt more comfortable approaching students with the evaluation tool (92% vs 43%)	None identified
Tracey et al., 2013 [39]	Nursing students	To evaluate the use of QR codes for facilitating self-directed learning in a nursing skills laboratory	QR codes were placed at strategic locations in a skills lab to allow students to watch video clips in order to help them learn a skill	To evaluate the activity, students were asked whether they found the use of QR codes to be helpful. “As expected, the response was overwhelmingly positive”. Students stated that using QR codes was “very helpful,” “wished there were more,” and were an “excellent source for learning hands-on topics.”	A few of the students did not have a smartphone or did not know how to use one
Traser et al., 2015 [40]	Medical students / Doctor of physical therapy students	To study student perceptions on the usefulness of QR codes as learning aids in a medical gross anatomy course	Question prompts and QR codes were tagged on cadaveric specimens to aid with learning	Students responded positively to the inclusion of QR codes in the gross anatomy laboratory. 56% stated they found the QR codes to be more helpful than traditional study-aids and the majority (89%) felt that QR codes helped them improve their learning of anatomy. A significant positive correlation existed between students’ usage of QR codes and their perception for enhancing learning of anatomy. 81% of students thought codes were accessible during self-directed study time	Student reluctance to use phones in the gross anatomy laboratory. Lack of adequate technology and complications with scanning QR codes or accessing the internet
Upton et al., 2017 [41]	Healthcare staff	To add a QR code to a patient-held immunotherapy alert card	Providing management algorithms via QR codes on patient-held immunotherapy alert cards for healthcare staff looking after patients with potential reactions to novel anti-cancer systemic drugs	Rationalised benefits include: Availability across all operating systems, quick access to information, cost-effective, small size, ability to save information accessed, monitoring of usage for audit	The use of a static QR code can cause problems if the web page is moved. Lack of familiarity with QR codes amongst staff. Staff not owning a smart phone
Zurmehly & Adams, 2017 [42]	Nursing students	To explore the use of QR codes in a lecture and measure subsequent student satisfaction and engagement	QR codes were included in a medical surgical lecture to reveal the answers for an exercise on ECG rhythm interpretation	All students reported that scanning QR codes was easy, 90% stated that they found the QR codes to be more helpful than traditional textbook pictures, and most (97%) felt that the QR codes helped improve their learning of cardiac rhythm strips. All of the students reported that they would most likely use QR codes in the future. In the course evaluation, students expressed gratitude to the faculty for the use of QR codes and for the creative use of technology for learning in the classroom	Some students did not have a smartphone or a phone that could download a QR code reader and were hesitant to indicate this

### Theme 1: increasing participant engagement

Nine articles discussed ways in which QR codes could be utilised to enhance participant engagement [20, 28–32, 36, 40, 42]. These can be broadly divided into those focusing on anatomy teaching, formative assessment, case-based learning and engagement with publications.

Three articles discussed the application of QR codes for anatomy teaching [32, 36, 40]. Mogali et al. reported how a medical student’s experience of an anatomy specimen museum could be enhanced by the use of QR codes [32] attached to specimens. This allowed further contextual information to be gained, such as annotated images and clinical histories. In this study, 78% of students agreed that QR codes were useful for learning and the majority of students agreed or strongly agreed that specimen information was easy to access via QR codes. Key benefits of QR codes cited by the author included its low-cost and adaptability in any learning environment. It also limits damage to specimens from actual handling. In a similar manner, Siderits et al. used QR codes to facilitate the distribution of, and enhance the educational content at, tumour board presentations [36].

Two studies used QR codes to aid learning through formative assessment [40, 42]. From questionnaire responses, over 80% of students reported that they found QR codes to be more helpful than traditional learning aids. However, despite this, neither study demonstrated a positive impact on student examination performance compared to the control group. There were high levels of total engagement in both cases and students using QR codes for gross anatomy learning, in particular, appreciated the receipt of immediate feedback. The authors also discuss how their use can act as a cost-effective self-assessment solution [40].

Lin et al. investigated the use of QR codes during a pharmacology course for third year nursing students in Taiwan [28, 29] and reported most participants held positive attitudes towards QR codes, with their utility for learning activities being cited as a key benefit. Lin noted that when used for case-based learning, a subset of students performed better in asking questions and discussions than they did prior to the introduction of QR codes.

Finally, an editorial by MacRae reported on the introduction of QR codes onto articles in the journal, Neurosurgery [30]. This has allowed linking to further multimedia information and easier dissemination by readers.

The majority of the aforementioned studies demonstrated how students found QR codes easy to use [20, 28, 29, 40, 42] and had a desire to continue using them in the future for classroom exercises or otherwise [20, 28, 29, 42].

Problems associated with implementation of QR codes for increasing engagement included: the requirement for internet access for full functionality [29, 42] and difficulties with downloading a QR code reader onto smartphones [40, 42].

For anatomy teaching, limitations were related to the sensitive information or images that may be contained within QR codes. Two studies therefore had to remind students that learning resources were for personal use only [32, 40]. Mogali et al. password protected PDF documents that were linked to QR codes to prevent amendments and printing [32]. Additionally, Traser et al. indicated that students were reluctant to bring their phones into a gross anatomy laboratory [40].

Interestingly, 47% of students in Lin’s study declined to trial the use of QR codes because of eyestrain from the use of cellular devices and distractions from work by surfing the internet [29]. Zurmehly et al. also noted that not all students had a smartphone [42]. This problem was overcome during the study through pairing students without a smartphone with those who did have one. Other limitations discussed included the need to ensure QR codes were large enough and not wrinkled from the surface they were applied to; both of which would make it harder for the scanner to read.

### Theme 2: Just-in-time (JIT) learning

Six articles discussed how QR codes can be used for JIT learning [25–27, 34, 39, 41]. This is a paradigm in which training is available on demand and can be accessed by staff when needed [43]. These applications can be broadly divided into those that allow reference to guidelines [26, 27, 34, 41] and those that contain information on how to perform a skill or use a piece of equipment [25, 27, 39]. For example, Tracey et al. discuss the recording of video clips demonstrating step-by-step procedures for wound care, medication administration and Foley catheter insertion [39]. Downer et al. discuss the use of QR codes for using equipment such as blood pressure machines [25].

Articles on the use of QR codes for referencing include checking plastic surgery and immune-related adverse event guidelines [26, 41], allowing nurses to view the management guidelines for falls [27] and accessing doctor’s phone numbers, in a labour room [34]. Rosario et al. demonstrated that QR codes provide high levels of functionality, usability and usefulness [34]. Gardiner et al. perceived that QR codes have a significant role in disseminating information to both healthcare staff and patients, with environmental and cost benefits because QR codes can ‘never run out’ [26].

The majority of articles containing JIT learning for skills or equipment-use, are linked to a video clip that the user could watch [25, 39]. Benefits associated with this included decreasing the downtime of students as they no longer had to wait for a facilitator to demonstrate [39] and the portability of the videos from access on a smart device [25].

A number of studies [25, 34, 39] have reported how healthcare staff find QR codes suitable for JIT learning as they are convenient and allow access to material in their own time and at a suitable pace for them. Interestingly, Tracey et al. noted how the response to the use of QR codes was overwhelmingly positive, particularly from the millennial generation [39].

Similar to other studies [29, 40], Jamu identified a reluctance to use devices in certain environments [27]. In this case, a clinical environment in front of patients. Additionally, Jamu noted a need for technical support in those with ‘little or no’ previous IT experience.

### Theme 3: simulation

Four articles discussed the adoption of QR codes to aid with simulation [21–24]. Damjanovic discussed the use of QR codes in creating a low-budget point-of-care ultrasound simulator [24]. QR codes were printed and stuck on an ultrasound phantom for practice. This provided a less resource intensive solution compared to radio frequency identification or near-field communication alternatives that require hardware. However, it was acknowledged that there is a time cost related to ensuring correct coding for corresponding video clips.

A novel use of QR codes in augmented reality (AR) integrated simulation training was reported by Carlson [22]. In this study, an average of five QR codes were used per scenario to act as a marker in order to trigger virtual images from AR software. Facilitators also had QR codes that could be scanned in order to validate a correct action. Perceptions related to this integrated simulation were rated positively, although no specific questions relating to the use of QR codes were asked. Users also felt that this particular application was a not a replacement for more traditional simulation teaching.

Two studies discuss QR code application in combination with gamification to allow for the identification of a simulated patient or medication or in order to identify the user involved [21, 23]. Problems were identified with their use during these studies. These included difficulties with the QR code scanner reading the code during a game [21] and, as previously discussed, difficulties in co-ordinating a reliable internet connection and downloading a QR code scanner [23].

### Theme 4: training support

Four articles explore how QR codes can be used to improve trainee experience, either through improvement in feedback methods [33, 37, 38] or by recording procedures undertaken in a log book [19].

Sobhani et al. [38] and Reynolds et al. [33] both investigated how feedback could be received through assigning an individual QR code to each student or resident that could then be scanned by a faculty member. In both cases, it was demonstrated that faculty found the QR-linked feedback form superior to the paper alternative.

Sobhani et al. demonstrated that faculty found the feedback method easier to understand and navigate, with faculty feeling more comfortable to approach students [38]. However, students from the study did not report increased efficacy to elicit feedback when compared to the paper form.

Reynolds findings from a mix of faculty and students indicated that the QR form provided improved educational benefits [33]. Overall, more evaluations were also submitted by residents over 6 months compared to paper assessments.

Snyder et al. compared the use of QR feedback forms at 15 family medicine clerkship sites across the United States to online or paper alternatives [37]. It was found that the QR feedback forms were associated with the highest usability score and took the shortest amount of time to prepare by students. Although, adoption of the QR feedback forms was challenged by wireless network limitations.

Avidan et al. discussed the use of QR codes for improving the logging of cases for anaesthetic trainees [19]. In this study, QR codes were generated by a computer system at the conclusion of each case containing information such as the date of surgery, type of surgery and type of anaesthesia. This was then scanned by a trainee using their smartphone to store the data in their own spreadsheet. Avidan et al. hypothesised that this method would reduce reliance on the IT department, whom trainees contacted to receive a list of all their cases at the end of their residency. It would also ensure that trainees did not come to the end of their residency and realise that they had not performed enough of a particular type of procedure, at which point the deficit would be harder to correct.

Evaluation was performed prior to the introduction of QR codes and then at 3 and 6 months after introduction. Residents rated that usability of the technology highly and most stated they would recommend the use of QR codes for case logging to colleagues. The overall rate of case logging had increased by over 45% when compared to before the introduction of QR codes.

## Discussion

This review has provided an overview of the diverse uses of QR codes in healthcare education. We reviewed 24 articles, published over the last 10 years, which demonstrated that the exploration of QR codes for healthcare education is still in its infancy. However, over half of the articles reviewed were published in the last 2 years, suggesting an increasing interest in the subject area.

The key benefits of using QR codes for healthcare education lie in the ability to provide timely, multimodal information in a cost-effective manner. Several studies demonstrated an improvement in engagement from students or faculty [19, 28, 29, 32, 33, 37, 38, 40, 42], which may be due to ease of use, availability and immediacy compared to other available methods of accessing information. Additional benefits lie in adaptability, simplicity of creation and potential environmental benefits of QR codes. In addition, the use of *dynamic* QR codes allows the creator to monitor useful metrics including: where the code was scanned, device used to scan and at what time the code was scanned. This could allow identification of topics that students are finding more difficult, or help in producing more tailored resources for students as a result [40].

In addition to the included articles, we identified three published conference abstracts that provided further examples of QR code utilisation for healthcare education. Following the introduction of QR codes to access a form to record procedural experiences by anaesthetic trainees, Singhapricha et al. demonstrated a 52% increase in the number of intubations and central venous access procedures logged, compared to the previous year [44]. Kee et al. have demonstrated that using QR codes in anaesthetic presentations help to enhance submissions by offering immediacy and raising the level of participation of the reader [45]. Additionally, a questionnaire distributed to members of the International Society for Medical Publication Professionals by McGrath and Fisher aimed to gain insight into the benefits of using QR codes for scientific poster presentations [46]. Suggested benefits included: increased audience engagement, wider data dissemination, enhanced poster metric tracking and increased capacity for multimedia presentation.

Our review has also highlighted some important considerations and potential challenges associated with the use of QR codes in healthcare education, particularly in a clinical setting.

Firstly, use of smartphones in the clinical environment to scan QR codes could be perceived as unprofessional by some patients. Thus, consideration needs to be given to the governance of introducing a QR code solution into a healthcare education setting. It is important to advise doctors, trainees and students on appropriate use of smartphones to scan QR codes in the clinical environment and to inform patients that smartphones are being used to support teaching. In addition, there are potential risks when using a smart device in certain settings, such as in an anatomy laboratory [40] where accidental or deliberate inappropriate sharing of sensitive (or patient identifiable) information could occur. Similarly, teachers need to ensure QR codes intended for healthcare professional education are secure and can only be accessed by the intended audience.

Secondly, several authors identified the need for a fast and reliable internet connection which can be a limitation in some clinical areas [23, 34]. Two studies overcame this, either by choosing ward areas with adequate Wi-Fi signal [27] or by boosting the existing hospital Wi-Fi [34]. However, these options may not be applicable to all institutions worldwide and more widespread QR use may require improvement in technological infrastructure.

Other problems related to an inability to scan QR codes, either due to difficulty with the scanner or the way in which the QR code was displayed. However, these are likely to be minor issues that can be resolved with further adoption of the technology. A couple of studies have also acknowledged that their applications for QR codes could have been succeeded by automated software to capture data. This would have negated the need for QR codes in the first place. Albeit these software solutions would have been more resource-intensive [19, 23].

Patient safety concerns could arise if healthcare professionals were using QR codes to access guidelines that were either out of date, no longer in use or had been moved from their original online location, as alluded to by Upton et al. [41]. This can be avoided be ensuring that any QR codes generated are *dynamic* rather than *static*. Dynamic codes (usually only available with a website subscription) allow the linked resource to be changed without the need to replace the original QR code. Static codes, however, require a new code to be generated if the location or nature of the resource changes. In addition, the more information encoded in a static code, the more complex the code appears, which can impede scanning success. However, the one advantage of using a static QR code is related to the fact that they contain all information within the code itself. Therefore, they can work effectively in areas of poor network connection. If dynamic QR codes are used to provide access to guidelines, it is imperative that responsibility is taken by the institution to ensure the links are kept up to date.

Although, some students or staff may have a lack of familiarity with QR codes, own a smartphone that can download a QR code reader, or own a smartphone at all, this is likely to change moving forwards. In 2018, 78% of the UK adult population owned a smartphone, up from 17% a decade earlier [47]. The millennial generation are also establishing themselves in the healthcare environment with more competence and self-sufficiency with technology than their predecessors. Nonetheless, before smartphones become ubiquitous, it may lead to challenges by disadvantaging students and staff from certain socio-economic backgrounds and therefore affect their subsequent attainment.

### Limitations

Literature surrounding the use of QR codes in healthcare education is still relatively scarce, however, the body of evidence is growing rapidly. The majority of articles included in this review use qualitative student perceptions rather than quantifiable data from questionnaires. Although this can limit subsequent analysis, it is a reasonable outcome to measure for gaining initial perceptions. In addition, a majority of the articles are from Western countries or countries with more developed healthcare systems. This therefore raises the question of whether findings are generalisable to other areas of the world. Similarly, the majority of articles focus on the use of QR codes for nursing students or staff, raising the question of whether findings are applicable to other members of a multidisciplinary healthcare team. Despite these limitations, the overall perceptions and challenges encountered from each included article are congruent to one another.

## Conclusion

In the healthcare sector, we are generally slower at embracing innovation and new technologies compared to other industries. This is sometimes rightly so, due to concerns around confidentiality, infrastructure, equality of access, patient safety and ethics, amongst other considerations. However, as with any other innovation, the uptake of QR code technology will depend on demonstrating effective benefit for patients and professionals along with a background change in social norms. Improvements in network infrastructure across healthcare institutions is also required to allow for more effective adoption. We need to be mindful, however, that not every facet of healthcare education requires a technological solution. There may be some instances or environments where using smart devices is less beneficial. Nonetheless, QR codes provide an exciting opportunity to excite and engage learners in ways we have not been able to, thus far. Potential uses are still being explored and benefits have already been demonstrated in a select number of use-cases. Table 3 summarises the author’s top tips from the review for using QR codes effectively in healthcare education.

**Table 3:** Top 10 tips for implementing QR codes in healthcare education

Environment	
1. Is the educational environment appropriate for QR code use?	
2. Are the QR codes suitably printed and positioned for readability by a scanner?	
IT issues 3. Is there a suitable network connection for smartphone use?	
4. Has the target audience access to a smartphone with a QR code reader available?	
Governance 5. Are the target audience aware of any probity or professionalism issues associated with the use of smartphones to scan QR codes?	
6. Will there be any unintended users of the code and what impact may this have? (e.g. misinterpretation, harm, negative public perception)?	
Logistics	
7. Should the QR codes be *dynamic* or *static* in nature?	
8. Should the codes include any branding (e.g. an institutional logo in the centre) to distinguish it from other commercial QR codes?	
Safety	
9. Has the correct resource been encoded onto each QR code in use?	
10. How will information on the QR code be kept up to date?	

Further work should focus on feasibility studies related to the use of QR codes in clinical environments and the perceptions of patients towards their use. Guidelines for protecting patients, trainees and staff also needs to be considered. Further studies investigating whether the use of this technology can have a significant positive impact on student performance would also be beneficial.

## Data Availability

All data generated or analysed during this study are included in this published article and its references.

## Abbreviations

AR: Augmented reality
CINAHL: Cumulative index to nursing and allied health literature
HBE: Health business elite
HMIC: Healthcare management information consortium
JIT: Just-in-time
MeSH: Medical subject headings
PRISMA: Preferred reporting items for systematic reviews and meta-analyses
QR: Quick response
